# Conflict of interest and risk of bias in systematic reviews on methylphenidate for attention-deficit hyperactivity disorder: a cross-sectional study

**DOI:** 10.1186/s13643-023-02342-x

**Published:** 2023-09-26

**Authors:** Alexandra Snellman, Stella Carlberg, Louise Olsson

**Affiliations:** 1 Centre for Assessment of Medical Technology in Örebro, Örebro University Hospital, Örebro, Sweden; 2 School of Medical Sciences, Örebro University, Örebro, Sweden; 3 Department of Surgery, Lindesberg Hospital, Lindesberg, Sweden

**Keywords:** Systematic review, Conflict of interest, Disclosure, Risk of bias

## Abstract

**Background:**

Systematic reviews (SRs) are pivotal to evidence-based medicine, yet there is limited research on conflicts of interest in SRs. Our aim was to investigate financial conflicts of interest and risk of bias (RoB) in SRs of a well-defined clinical topic.

**Methods:**

A librarian searched Medline, Cochrane Library, Embase, and PsycINFO for SRs investigating the effect of methylphenidate on ADHD in December 2020. The selection process adhered to the PRISMA guidelines. Two blinded reviewers independently searched open websites, including other publications, for information on financial conflicts of interest of all authors of the included SRs. A time limit of 3 years before or after the index SR was adopted. Declarations on conflict of interest were extracted from the included SRs for comparison. ROBIS was used for RoB assessment.

**Results:**

Out of 44 SRs included, 15 (34%) declared conflict of interest, 27 (61%) did not, and a declaration of conflict of interest was missing for 2 (5%). On open websites, conflict of interest was found for at least one author of 23 (52%) SRs: disclosed in 15 (34%) and not disclosed in 8 (18%) SRs.

Seven (16%) SRs had low, 36 (82%) had high, and 1 (2%) had unclear RoB. Among SRs with financial conflict of interest found in open sources, 6/22 (27%) had low RoB compared to 1/21 (5%) if no such conflict of interest was identified. Among SRs with financial conflict of interest identified, 1/6 (17%) at low RoB did not disclose their conflict of interest, whereas the corresponding proportion among SRs at high RoB was 7/16 (44%).

Eight (18%) SRs presented conflict of interest disclosed in the included primary studies. Four of them (50%) had low RoB, compared to 3/36 (8%) for SRs not reporting on this aspect.

**Conclusion:**

Financial conflict of interest was underreported in 18% of the SRs using our reference standard, and overall it was present for every second SR. This group embraced both SRs at low RoB disclosing conflict of interest and SRs at high RoB not disclosing their conflict of interest. Further studies to explore this heterogeneity are warranted.

**Supplementary Information:**

The online version contains supplementary material available at 10.1186/s13643-023-02342-x.

## Introduction

Systematic reviews (SRs) provide guidance for policymakers and practitioners. It is therefore of vital importance that they are conducted in a way that foster reliable results [1]. Any publication presented as a SR should include detailed, systematic, and transparent means of gathering, appraising, and synthesizing evidence to answer a well-defined question. Until now, the main focus has been on the methodology and the assessment of risk of bias, and only limited attention has been paid to any possible impact on the results on a group level associated with conflict of interest among the authors of SRs [2].

Conflicts of interest in SRs may emanate from two levels: from the authors of the SR itself and from the included primary studies. Conflicts of interest may bias the results of a SR in several ways, including through the selection of primary studies, assessment of risk of bias, choice of outcome, presentation of extracted data, analysis, and phrasing of the overall conclusion. Financial conflict of interest is the most commonly recognized source of bias, but the phenomenon also involves professional, intellectual, or advocatory aspects [3]. Conflict of interest is disclosed by individual authors and may be perceived differently, especially regarding subtle delimitations, and policies may vary between journals [3, 4], in spite of general recommendations [5]. The accuracy of conflict of interest disclosed by the authors and presented in the SRs is thus a relevant research assignment, given the impact of these publications on top of the pyramid of evidence.

The aim of this cross-sectional study was to compare conflict of interest disclosed by authors of SRs of a well-defined clinical topic *versus* information on financial conflict of interest found in open Internet sources, in relation to risk of bias of the SRs.

## Methods

In order to delimit the study, it was decided to focus on SRs reporting on the effect of methylphenidate for attention-deficit hyperactivity disorder (ADHD). This decision was favored by the potential inclusion of a large number of eligible SRs and the absence of any professional connections to the field among the investigators.

### Identification of systematic reviews

The methodology of SRs, including the PRISMA guidelines [6], was applied to identify the study material. Firstly, the databases Medline, Cochrane Library, Embase, and PsycINFO were searched by a medical information specialist at the Medical Library, Örebro University, for SRs on methylphenidate and ADHD from inception until 2020 December 18 (Additional file 1). Secondly, two reviewers (AS, LO) independently assessed the relevance of the identified publications, initially by screening the titles and abstracts, and then by reading the downloaded full-text versions of the selected publications. Only SRs published in English were eligible, as most SRs on this topic are published in English. The inclusion criteria embraced SRs on the treatment effects and/or adverse events of methylphenidate compared to placebo or any other pharmacological or non-pharmacological intervention among individuals diagnosed with ADHD. Publications presenting a meta-analysis but that were not classified as a SR by the authors themselves were not eligible. Any disagreement between the reviewers for inclusion was resolved in consensus.

### Financial conflicts of interest retrieved from online resources

In order to generate a reference standard, two reviewers (AS, SC) independently searched open websites for information on potential financial conflict of interest for all authors of all the included SRs. One of the reviewers (SC) was blinded to all other aspects of the study at this stage, whereas the other reviewer (AS) was blinded to the disclosures on conflict of interest in the SRs.

All the searches on the Internet followed the same routine. It always started with the official websites related to the affiliations of the authors as presented in each SR. The homepages of, e.g., universities often present their researchers, and publications lists or presentations of research projects were used to confirm the identity of each author. Authors that had both a common name and were not affiliated to an organization that provided a publication list at their website were difficult to assess. In case the identity of an author not could be confirmed with full certainty, no further searches for that specific author were undertaken, and the search moved on to the next author of the SR. For authors, whose identity was confirmed, a search for relevant data on financial conflict of interest followed. Links to collaborations and projects provided at their homepages were opened for this purpose. The procedure was repeated in the same way on LinkedIn and ResearchGate, i.e., the identity of the authors had to be confirmed, all relevant links were opened, and data related to financial conflict of interest were retrieved. Any findings on employments or assignments, grants, or other types of funding from industry were of interest. In each case, it was ensured that the benefits originated from a company providing products for treatment of ADHD. The amount of money involved was rarely presented, and the judgments relied on descriptions of the purpose of the compensations, e.g., lectures or any consultancy. Single events, like a lunch or a dinner (~ 50–100 €), did not count as a conflict of interest in this study. A time limit of 3 years between any financial conflict of interest and the publication of the SR was arbitrarily applied with the intention of ensuring that the conflict of interest would still be relevant to the author.

Relevant publications of each author published within 3 years prior to or after the index SR were then searched on PubMed, as the publications lists provided on authors’ homepages are not always complete. The full-text versions of the publications were examined for information on disclosures of conflicts of interest, and any pertinent data were retrieved. If the author had published on a wide range of topics, only those related to the index SR were of interest. If no information was found for a specific author, or if it was uncertain, this was coded as no conflict of interest.

In the next step, based on the findings of financial conflict of interest among the authors, each SR was dichotomized into the presence of financial conflict of interest or not. No attempt was made to differentiate the impact of financial conflict of interest based on, e.g., the position of the author or the proportion of authors for whom financial conflict of interest had been identified. Even if a financial conflict of interest had been identified for merely one out of all the authors, the SR itself was categorized as being exposed to a conflict of interest. Likewise, it was not possible to differentiate between larger or smaller sums involved. The reason for this straightforward dichotomization was the lack of any detailed insight into the working relations between the authors, and, conceptually, conflicts of interest are not associated with any specific sum of money.

As soon as the two reviewers had completed their examinations of all authors of the SRs, they compared notes and discussed their observations. The findings were inconsistent for seven SRs initially, prompting the reviewers to repeat their searches and then make a joint decision. Overall, the guiding principle for the retrieval of online data on financial conflict of interest was assurance and to err on the right side of caution. Once all the SRs had been categorized as exposed or non-exposed to financial conflict of interest, the findings were locked, and they remained unchanged throughout the study.

### Risk of bias assessment

The included SRs were assessed for risk of bias (RoB) using ROBIS [7]. This tool is organized in four domains each focusing on a specific aspect of RoB; study eligibility criteria, identification and selection of studies, data collection and study appraisal, and synthesis and findings. A thorough review of any associated protocols, or registration documents, constitutes an integral part of ROBIS. Ultimately, the ROBIS tool yields a comprehensive summary assessment of the RoB. If one domain is associated with a high RoB, the other domains cannot compensate for this already compromised trustworthiness, and the summarizing assessment of that particular SR cannot be any better than high RoB [7]. We took advantage of this design of the tool and adopted an “early-stop algorithm,” i.e., one reviewer assessed all the domains of the SRs, whereas the second assessed the domains consecutively until a high risk of bias was encountered (early stop). In this way, two reviewers (AS, LO) independently assessed the RoB of all the included SRs. If at least two domains were classified as “unclear,” the SR itself was classified as unclear. Finally, the RoB assessments were compared between the reviewers, and any disagreements were resolved through discussions until consensus was reached. The assessments were conducted minimum 3 months after the Internet searches, in order to attain a substantial washout period for one of the investigators (AS). Finally, SRs categorized as either at high or low risk of bias were cross-tabulated with SRs categorized as either having or lacking financial conflict of interest identified through open websites.

### Data extraction from the SRs

Data from the SRs were extracted by one reviewer and cross-checked by another. This comprised basic characteristics such as the number of primary studies, the number of participants included in each SR, the reported outcome of methylphenidate categorized as therapeutic treatment effects and/or adverse events, and the type of analysis conducted (meta-analysis, network meta-analysis, meta-regression, narrative analysis). Each included SR was also searched for information on conflict of interest among the included primary studies.

Data on whether this undertaking had been completed or not, and, if completed, the proportion of the included primary studies disclosing conflicts of interest, were extracted.

Statements on conflict of interest disclosed by the authors of the SRs themselves were retrieved and categorized as “Yes” (the authors declared the presence of conflicts of interest), “No” (the authors declared there were no conflicts of interest), or “Information missing” (there was no declaration on conflicts of interest for that SR).

### Statistics and ethics

Percentages of SRs with financial conflict of interest and of the assessments of risk of bias were determined. Differences in the proportions of SRs with financial conflict of interest identified in open sources versus at high and low RoB were tested using Fisher’s exact test. A *p*-value < 0.05 was considered significant. The study involved no health-related data on patients, and any ethical approval was not required.

## Results

**Fig. 1:**
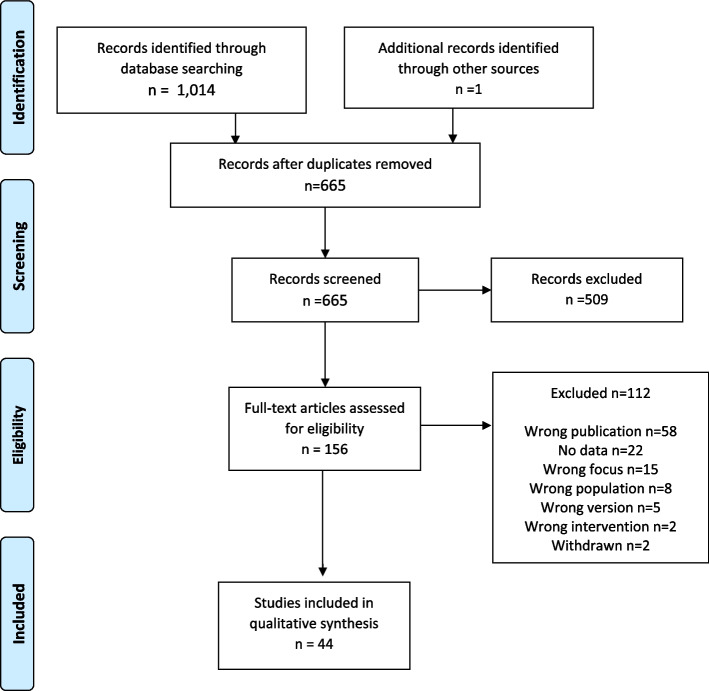
Study flow chart

The literature search yielded 665 unique hits, and 156 publications were selected for full-text reading (Fig. 1). In all, 112 publications were excluded with specific reasons (Additional file 2) and 44 SRs included (Additional file 3).

The first SRs included were published in 2008, and the number of included SR published per calendar year since then was median 2.5 (range 0–8) (Table 1). The number of included primary studies per SR was median 20.5 (range 4–269). A majority of 32 (73%) of the SRs included randomized controlled studies only, whereas 4 (9%) included non-randomized controlled studies and 8 (18%) primary studies of mixed design. For the analysis, 26 (59%) SRs presented a meta-analysis, eight (18%) a narrative analysis, six (14%) a network meta-analysis, two meta-regressions, and two SRs presented both a meta-analysis and a narrative analysis. Therapeutic effects of methylphenidate were presented in 18 (41%) systematic reviews, 15 (34%) reported on both treatment and adverse effects, and 11 (25%) reported on adverse effects of methylphenidate only.

**Table 1:** Basic characteristics of the included systematic reviews (*n* = 44)

**SR**	**Publication year**	**SR ID in Additional file** 3	**Primary studies (*n*)/participants (*n*)**	**Study design of primary studies**^a^	**Type of analysis**^b^	**Therapeutic outcomes reported**	**Adverse effects reported**
1	2021	Candido [5]	10/497	RCT	MA and narrative	x	x
2		Rodrigues [35]	25/1257	Mixed	MA	x	x
3	2020	Elliot [13]	64/12,423	RCT	Network MA	x	
4		Koren [21]	4/2831	NRCT	MA		x
5	2019	Faraone [14]	35/5685	Mixed	MA		x
6		Kortekaas [22]	34/2233	Mixed	MA	x	
7		Liu [26]	8/4,221,929	NRCT	MA		x
8		Stuhec [38]	20/5428	RCT	MA	x	
9		Sun [39]	8/242	RCT	MA	x	
10		Villas-Boas [43]	5/NR	RCT	Narrative	x	x
11		Yan [44]	18/778	RCT	MA	x	
12	2018	Cortese [11]	133/15,430	RCT	MA	x	x
13		Lenzi [24]	21/4376	RCT	MA	x	
14		Liang [25]	22/46,107	Mixed	MA		x
15		Padilha [30]	33/3493	RCT	Network MA	x	x
16		Pievsky [31]	24/832	RCT	MA	x	
17		Pozzi [32]	45/8283	RCT	MA		x
18		Storebo [37]	269/ > 2 millions	NRCT	MA and narrative		x
19		Tarant [41]	12/≈ 434	RCT	Narrative	x	x
20	2017	Carpentier [6]	8/913	RCT	Narrative	x	x
21		Catala-Lopez [8]	190/26,114	RCT	Network MA	x	x
22		Hennissen [18]	18/6037	Mixed	MA		x
23		Holmskov [19]	61/3564	RCT	MA		x
24		Joseph [20]	36/NR	RCT	Network MA		x
25		Liu [27]	11/3153	RCT	MA	x	x
26	2016	Bushe [4]	14/≈ 2879	RCT	Network MA	x	x
27		Chan [9]	17/2668	Mixed	MA	x	
28		Rezaei [34]	11/2772	RCT	MA	x	
29		Tamminga [40]	50/1611	RCT	Metaregression	x	
30	2015	Barkla [1]	20/NR	Mixed	Narrative	x	x
31		Coughlin [12]	23/2959	RCT	Metaregression	x	
32	2014	Bellino [2]	45/5923	Mixed	Narrative	x	x
33		Coghill [10]	60/1994	RCT	MA	x	
34		Maneeton [29]	4/146	RCT	MA	x	
35		Roskell [36]	32/NR	RCT	Network MA	x	
36	2013	Castells [7]	12/2496	RCT	MA		x
37		Prasad [33]	14/2110	RCT	MA	x	
38	2012	Lv [28]	8/478	RCT	Narrative	x	x
39	2011	Hanwella [17]	9/2762	RCT	MA	x	
40	2009	Bloch [3]	9/447	RCT	MA	x	x
41		Godfrey [16]	26/811	RCT	Narrative		x
42		Lan [23]	34/3167	RCT	MA	x	
43	2008	Ghuman [15]	20/NR	NRCT	Narrative	x	x
44		Van der Oord [42]	24/1482	RCT	MA	x	

^a^*RCT* only randomized controlled studies, *NRCT* only non-randomized studies, *Mixed* RCT and NRCT

^b^*MA* meta-analysis; direct comparisons of interventions, *Network MA* indirect comparisons across trials based on a common comparator

### Financial conflict of interest

A disclosure on conflict of interest was presented in 42 (95%) SRs and thus missing in 2 cases (Table 2). In 15/44 (34%) SRs, authors declared the presence of financial conflicts of interest, and these disclosures were confirmed in all cases. In another 8/44 (18%) SRs, authors declared no conflicts of interest but discordant information was available online as outlined above. In all, 23/44 (52%) of the SRs had financial conflicts of interest identified. Among these, the conflicts of interest were disclosed in 15/23 (65%) SRs. No conflict of interest was declared by authors of 27 SRs, and the information was consistent with online findings for 19/27 (70%) of them (Table 2).

**Table 2:** Conflict of interest declared by authors of the systematic reviews vs identified in online resources (*n* = 44). Values in parentheses are column percentages

		**Financial conflict of interest in open online resources**		
		Yes	No	Total
**Financial conflict of interest declared by authors**	Yes	15 (65)	0	15 (34)
	No	8 (35)	19 (90)	27 (61)
	Missing	0	2 (10)	2 (5)
	Total	23 (100)	21 (100)	44 (100)

### Risk of bias assessment

**Fig. 2:**
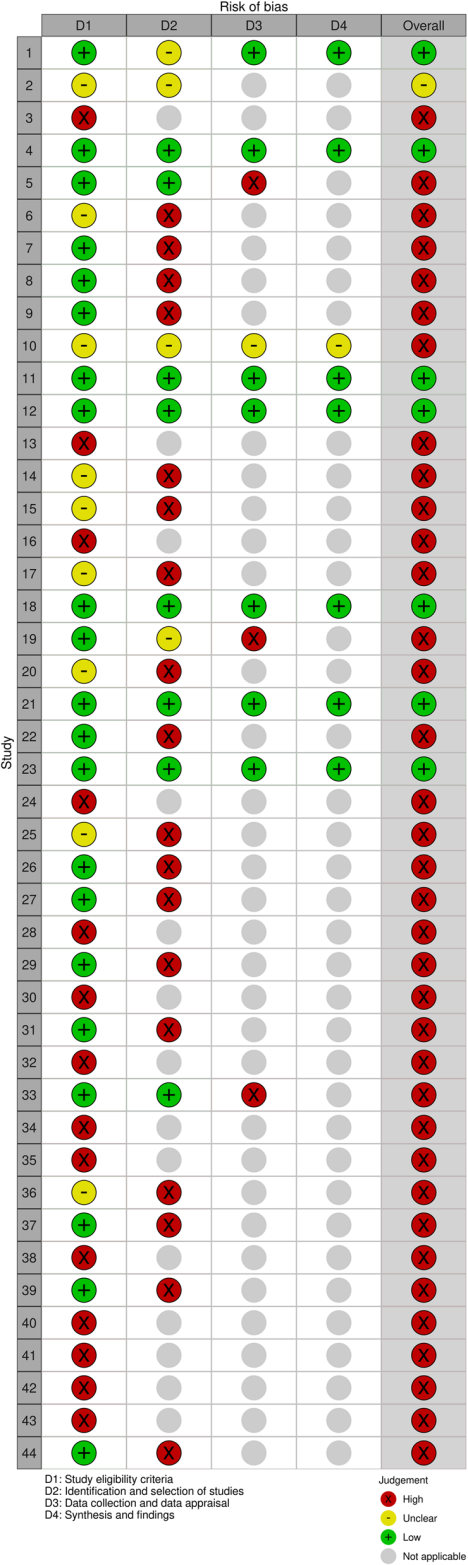
Risk of bias assessment using ROBIS of the included systematic reviews (*n* = 44)

Two reviewers working independently initially had divergent RoB assessments for five SRs, which was resolved in consensus. In total, seven (16%) SRs were found to be at low RoB (Fig. 2), all published from 2017 and onwards. One SR had two domains judged as “unclear” (“study eligibility criteria” and “selection of studies”), and the summary for this SR was hence “unclear” RoB. The remaining 36 (82%) SRs were found to be at high risk of bias, and the reasons were shortcomings in study eligibility criteria (domain 1) in 12/44 (27%), in identification and selection of studies (domain 2) in 20/44 (45%), in data collection and study appraisal (domain 3) in 3/44 (7%), and in synthesis and findings (domain 4) in 1/44 (2%) SRs (Fig. 2). The descriptions of the study eligibility criteria in 13 SRs with shortcomings in domain 1 (10 with high and 1 with unclear RoB) were carefully reviewed. No study protocol was found for nine of them despite significant searches, and for the remaining four SRs, the protocols conveyed the same incomplete information as the SR themselves.

In all, 43 SRs were categorized as either at high or low RoB. One (2%) SR had low risk of bias and no financial conflict of interest identified on open websites, and 16/43 (37%) SRs had high risk of bias and financial conflict of interest identified (Table 3). The proportion of SRs at low RoB among SRs with financial conflict of interest found on open Internet sources was 6/22 (27%) vs 1/21 (5%) among SRs with no such conflict of interest found (Fisher’s exact test 0.09; *p* > 0.05). Among SRs with financial conflict of interest identified in open sources, 1/6 (17%) at low RoB had not disclosed this information in their publication in contrast to 7/16 (44%) SRs at high RoB.

**Table 3:** Risk of bias of systematic reviews according to ROBIS *vs* conflict of interest identified in online resources (*n* = 43). Values in parentheses are column percentages

		**Financial conflict of interest in open online resources**		
		Yes	No	Total
**Risk of bias**^a^	High	16 (73)	20 (95)	36 (84)
	Low	6 (27)	1 (5)	7 (16)
	Total	22 (100)	21 (100)	43 (100)

^a^One systematic review with unclear risk of bias was not included in the table

### Financial conflict of interest in the included primary studies

Eight (18%) SRs presented data on financial conflict of interest as disclosed in the included primary studies, and they were evenly distributed between SRs with and without financial conflict of interest identified on open websites (Table 4). The total number of SRs with no financial conflict of interest identified, and some attention paid to financial conflict of interest of the included primary studies, was thus 4/44 (9%). The proportion of included primary studies that declared financial conflict of interest ranged from 35 to 89% in the eight SRs that provided this information. In four of them, it was discussed whether the primary studies were biased by the presence of financial conflict of interest, and one of them looked into potential consequences by conducting a sensitivity analysis. The remaining four SRs did not analyze, nor discuss the matter any further.

**Table 4:** Systematic reviews reporting conflict of interest of the included primary studies *vs* conflict of interest of the systematic reviews identified in online resources (*n* = 44). Values in parentheses are column percentages

		**Financial conflict of interest in open online resources**		
		Yes	No	Total
**SR reporting conflict of interest of the included primary studies**	Yes	4 (17)	4 (19)	8 (18)
	No	19 (83)	17 (81)	36 (82)
	Total	23 (100)	21 (100)	44 (100)

Out of the 8 SRs that presented data on conflict of interest in the included primary studies, 4 (50%) had a low RoB, whereas the corresponding proportion among 36 SRs that did not present this information was 3 (8%) SRs.

## Discussion

In this study using open websites as the reference standard, an underreporting of financial conflict of interest was observed in 18% of the SRs. In all, half of the SRs were associated with financial conflict of interest and more than 80% at high RoB. Among SRs with financial conflict of interest found in online resources, 27% had low RoB compared to 5% of SR with no such conflict of interest identified. A larger proportion of SRs at low risk of bias disclosed their conflict of interest compared to SRs at high RoB.

To the best of our knowledge, we have not encountered any other study adopting a procedure similar to ours for investigating the accuracy of disclosures of financial conflict of interest in SRs, despite the simplicity of accessing in practice open websites controlled by the authors themselves. A somewhat comparable approach though was used in a systematic meta-review on new drugs for the treatment of arthritis published already in 2009 [8]. In 281 identified, mainly narrative, reviews, it was noticed that the authors of 44 (16%) of them had published multiple reviews within a short time period of time, but conflict of interest was disclosed only in one of the reviews. Likewise, the authors of 62 (22%) reviews had been involved in at least one clinical trial reasonably close in time but did not disclose this conflict of interest in their reviews. The extent of underreporting of financial conflict of interest observed in the present sample of SRs may therefore not deviate much from previous studies. Concerning actual disclosure of conflicts of interest, we found this was the case for one third of the included SRs, and this matches previous findings. Hakoum et al. searched 119 core clinical journals and the Cochrane Database of Systematic Reviews for SRs published in 2015 and found 30% of the non-Cochrane, and 49% of the Cochrane reviews disclosed conflicts of interest [3]. An investigation of SRs published up to 2013 on sugar-sweetened beverages and weight gain or obesity found 35% of the SRs disclosed financial conflict of interest [9].

An overwhelming majority (82%) of the SRs were at high RoB in the present sample. Most common reasons were suboptimal identification and selection of studies in almost half and unsatisfactory definitions of study eligibility criteria in more than one fourth of the included SRs. However unsatisfactory this may be, it still seems to be an improvement compared to the merely 5% of SRs explaining the search criteria explicitly, or using a criterion-based selection of studies, as was reported by Roundtree in 2009 [8]. Another cross-sectional study, published in 2020, investigated 78 SR/meta-analyses in the field of bariatrics and found 78% were at high risk of bias [10], and a recent investigation of 48 SRs on COVID-19 using the ROBIS tool reported 5/48 (10%) were at low risk for bias [11]. Such large proportions of SRs at high risk of bias challenge the general recommendation of always approaching a new clinical topic by reading the SRs first. It also highlights the importance of training to spot methodological shortcomings. A number of tools for the assessment of risk of bias/study quality have been developed over time, e.g., the Cook et al. score, AMSTAR, AMSTAR-2, and lately ROBIS introduced in 2016 [7, 12–14]. Our endeavor to optimize the risk of bias assessment of SRs by implementing an early-stop algorithm of ROBIS for one of the reviewers was rewarding. It may possibly facilitate further investigations on risk of bias in a larger number of SRs on various topics or even be helpful to readers of SRs in general.

Interestingly, a larger proportion of SRs *with* financial conflict of interest had low RoB compared to those without such conflict of interest in this sample (Table 3). This finding contrasts with Hansen et al. who reported that SRs with conflict of interest tend to be of lower methodological quality [15]. We believe our observation deserves further attention, in particular as authors of SRs at low RoB were more likely to disclose their financial conflict of interest in the present study. In our understanding, this underlines the importance of reporting financial conflicts of interest and the traditional aspects of RoB separately, as they represent different phenomena [16–19].

A troublesome finding of this study, in our opinion, was the low frequency of SRs that presented data on financial conflict of interest among the included primary (8/44; 18%). However, according to a recent study on 250 meta-analyses, author-industry financial ties of the included trials were reported in only 1% of non-Cochrane and 44% of Cochrane meta-analyses [20]. Conflict of interest among the authors of primary studies may be associated with, e.g., tactical choices on control groups or comparative interventions [21], and we understand that information on competing interests related to the included primary studies is essential to all the end-users of SRs. The Cochrane collaboration is very clear on this aspect in the latest version of their handbook [16], whereas the updated PRISMA guidelines focus on competing interests among the authors of the SR itself [22].

The recent update of Cochrane’s policy also stresses the importance of non-financial types of conflict of interest [16], and a limitation of this study may be the focus on merely financial conflict of interest. In this case, individual professional or intellectual conflict of interest may have been valid [3]. Another drawback is the moderate number of SRs in the study, but our search for eligible SRs began from inception, and two independent reviewers read a substantial number of publications in full-text to ensure the inclusion of all relevant SRs. A merit of the study is the search for financial conflict of interest carried out by two independent, blinded reviewers employing a consistent mode of procedure for all the searches on open websites controlled by the authors/researchers themselves. Common names turned out to be an underestimated problem, especially if there was no English version of the homepages, and information on some authors, probably at the beginning of their career, was more difficult to retrieve. The searches also embraced other publications of the authors published within 3 years prior to or after the index SR. The information on authors’ declarations of conflict of interest in other publications was mostly supplementary and confirmatory but in a few cases also critical. Nevertheless, it cannot be entirely ruled out that some SRs might inaccurately have been classified as lacking financial conflict of interest. On the other hand, the confirmatory findings for 15 SRs whose authors’ disclosed conflict of interest reinforces the validity of the searches. Another strength of our study is a conscientious assessment of risk of bias of all the included SRs and an innovative way of using the ROBIS tool.

Several research opportunities for further exploration were noted. We interpret the findings on the SRs with financial conflict of interest identified to fall into three main categories—SRs at low risk of bias principally disclosing their conflict of interest and SRs at high risk of bias either disclosing or not disclosing financial conflict of interest. The findings and conclusions of the last group of SRs are obviously of particular interest. For primary studies, it has been shown that undeclared, as opposed to declared, financial conflict of interest are more likely to be associated with positive recommendations [18]. Another question is whether the proportion of authors with financial conflict of interest, and the scope of their interest, is of importance. This question has practical implications. If the impact of one author, as opposed to all authors, with financial conflict of interest is the same, an early-stop algorithm could be adopted for this aspect of assessment as well. However, one dilemma we noticed in this study was the extensive complexity of the SRs—the disclosures among authors often involved several companies, and the SRs often assessed multiple interventions. Without any detailed knowledge of the field as such, we understand it would be almost impossible to determine any common direction of the financial conflict of interest among the authors of a specific SR. The inclusion criteria of SRs for such a study would need to be quite strict. Nevertheless, a study on systematic reviews on neuraminidase inhibitors demonstrated that reviewers with conflict of interest were more likely to present favorable results [23].

In summary, this study found that financial conflicts of interest in SRs are underreported compared to information available on open websites. On the other hand, SRs with financial conflict of interest exhibited a higher proportion at low RoB, compared to SRs without such conflict of interest. We conclude that SRs with financial conflict of interest encompass several distinct subgroups, calling for further urgent investigations.

### Supplementary Information

**Additional file 1.** Search strategy.

**Additional file 2.** Excluded publications.

**Additional file 3.** Systematic reviews included.

## References

[1] World Health Organisation. Enhancing WHO’s standard guideline development methods 2019. Available from: https://www.who.int/news/item/28-01-2019-enhancing-who-s-standard-guideline-development-methods. [cited 2023 June, 21].

[2] Hansen C, Lundh A, Rasmussen K, Hróbjartsson A. Financial conflicts of interest in systematic reviews: associations with results, conclusions, and methodological quality. Cochrane Database of Systematic Reviews. 2019;8:Art. No.: MR000047. 10.1002/14651858.MR000047.pub2. Accessed 13 Sept 2023. 10.1002/14651858.MR000047.pub2 PMC7040976 31425611

[3] Hakoum MB, Anouti S, Al-Gibbawi M, Abou-Jaoude EA, Hasbani DJ, Lopes LC (2016). Reporting of financial and non-financial conflicts of interest by authors of systematic reviews: a methodological survey. BMJ Open.

[4] Shawwa K, Kallas R, Koujanian S, Agarwal A, Neumann I, Alexander P (2016). Requirements of clinical journals for authors' disclosure of financial and non-financial conflicts of interest: a cross sectional study. PLoS ONE.

[5] International Committee of Medical Journal Editors. Disclosure of Interest 2021. Available from: https://www.icmje.org/disclosure-of-interest/. [cited 2023 June, 21].

[6] Page MJ, McKenzie JE, Bossuyt PM, Boutron I, Hoffmann TC, Mulrow CD (2021). The PRISMA 2020 statement: an updated guideline for reporting systematic reviews. BMJ.

[7] Whiting P, Savovic J, Higgins JP, Caldwell DM, Reeves BC, Shea B (2016). ROBIS: a new tool to assess risk of bias in systematic reviews was developed. J Clin Epidemiol.

[8] Roundtree AK, Kallen MA, Lopez-Olivo MA, Kimmel B, Skidmore B, Ortiz Z (2009). Poor reporting of search strategy and conflict of interest in over 250 narrative and systematic reviews of two biologic agents in arthritis: a systematic review. J Clin Epidemiol.

[9] Bes-Rastrollo M, Schulze MB, Ruiz-Canela M, Martinez-Gonzalez MA. Financial conflicts of interest and reporting bias regarding the association between sugar-sweetened beverages and weight gain: a systematic review of systematic reviews. PLoS Med. 2013;10(12):e1001578; dicsussion e. 10.1371/journal.pmed.1001578 PMC3876974 24391479

[10] Storman M, Storman D, Jasinska KW, Swierz MJ, Bala MM (2020). The quality of systematic reviews/meta-analyses published in the field of bariatrics: A cross-sectional systematic survey using AMSTAR 2 and ROBIS. Obes Rev.

[11] Dang A MS, Rao P JMV, Sri Gurram N, Digijarala S, Dang S and Vallish B.N. A critical appraisal of the risk of bias in systematic reviews and metaanalyses pertaining to COVID-19 Coronaviruses. 2022;3(2):52–8.

[12] Cook DJ, Sackett DL, Spitzer WO (1995). Methodologic guidelines for systematic reviews of randomized control trials in health care from the Potsdam Consultation on Meta-Analysis. J Clin Epidemiol.

[13] Shea BJ, Grimshaw JM, Wells GA, Boers M, Andersson N, Hamel C (2007). Development of AMSTAR: a measurement tool to assess the methodological quality of systematic reviews. BMC Med Res Methodol.

[14] Shea BJ, Reeves BC, Wells G, Thuku M, Hamel C, Moran J (2017). AMSTAR 2: a critical appraisal tool for systematic reviews that include randomised or non-randomised studies of healthcare interventions, or both. BMJ.

[15] Hansen C, Lundh A, Rasmussen K, Hrobjartsson A. Financial conflicts of interest in systematic reviews: associations with results, conclusions, and methodological quality. Cochrane Database Syst Rev. 2019;8(8):MR000047. 10.1002/14651858.MR000047.pub2 PMC7040976 31425611

[16] Cochrane Handbook for Systematic Reviews of Interventions, Cochrane; 2022. Available from: https://training.cochrane.org/handbook/current/chapter-07. [cited 2023 June, 21].

[17] Bero LA. Why the Cochrane risk of bias tool should include funding source as a standard item. Cochrane Database Syst Rev. 2013(12):ED000075. 10.1002/14651858.ED000075. 10.1002/14651858.ED000075 PMC10898502 24575439

[18] Patel SV, Yu D, Elsolh B, Goldacre BM, Nash GM (2018). Assessment of conflicts of interest in robotic surgical studies: validating author’s declarations with the open payments database. Ann Surg.

[19] Sterne JA. Why the Cochrane risk of bias tool should not include funding source as a standard item. Cochrane Database Syst Rev. 2013;(12):ED000076. 10.1002/14651858.ED000076. 10.1002/14651858.ED000076 PMC10897924 24575440

[20] Turner K, Carboni-Jimenez A, Benea C, Elder K, Levis B, Boruff J (2020). Reporting of drug trial funding sources and author financial conflicts of interest in Cochrane and non-Cochrane meta-analyses: a cross-sectional study. BMJ Open.

[21] Dunn AG, Mandl KD, Coiera E, Bourgeois FT (2013). The effects of industry sponsorship on comparator selection in trial registrations for neuropsychiatric conditions in children. PLoS ONE.

[22] Page MJ, Moher D, Bossuyt PM, Boutron I, Hoffmann TC, Mulrow CD (2021). PRISMA 2020 explanation and elaboration: updated guidance and exemplars for reporting systematic reviews. BMJ.

[23] Dunn AG, Arachi D, Hudgins J, Tsafnat G, Coiera E, Bourgeois FT (2014). Financial conflicts of interest and conclusions about neuraminidase inhibitors for influenza: an analysis of systematic reviews. Ann Intern Med.

