# Supplementary material for: Conflict of interest and risk of bias in systematic reviews on methylphenidate for attention-deficit hyperactivity disorder: a cross-sectional study
Source: Syst Rev. 2023 Sep 26;12:175. doi: 10.1186/s13643-023-02342-x (PMC10521496; doi:10.1186/s13643-023-02342-x)
Supplement: Supplementary file 1 — Additional file 1. Search strategy. [file 13643_2023_2342_MOESM1_ESM.docx]

Additional file 1 Search strategy for identifying relevant systematic reviews

**Medline via Ovid 2020-12-18**

|  | | | Number of publications |
| --- | --- | --- | --- |
| Attention Deficit Hyperactivity Disorder | | | |
|  | 1. | attention deficit disorder with hyperactivity/ | 29,336 |
|  | 2. | (Attention Deficit Disorder* with Hyperactivity or Attention Deficit Hyperactivity Disorder* or Attention Deficit Disorder* or ADHD or ADDH).ti,ab,kf. | 34,278 |
|  | 3. | 1 or 2 | 41,200 |
| Methylphenidate | | | |
|  | 4. | methylphenidate/ or dexmethylphenidate hydrochloride/ | 7,223 |
|  | 5. | (dextroamphetamine or dexamphetamine or dextroamfetamine or dexamfetamine or dexmethylphenidate or adhansia or 'alpha phenyl 2 piperidineacetic acid methyl ester' or 'alpha phenyl alpha acetic acid methyl ester' or aptensio or attenta or 'c 4311-b' or 'c4311-b' or 'c4311b' or centedrin or concerta or cotempla or 'd erythro methyl phenidylacetate' or daytrana or difumenil or 'dl erythro methyl phenidylacetate' or methylphenidate or jornay or medanef or medicebran or medikinet or mefinad or metadate or 'methyl 2 phenyl 2 piperid 2 ylacetate' or 'methyl phenidate' or methylfenidaat or methylfenidate or methylin or methylphenidylacetate or methylphenindate or methylphenydate or methypatch or penid or 'phenidyl hydrochloride' or phenidylate or quasym or quillichew or quillivant or rilatine or ritalin or 'ritalin sr' or ritalina or ritaline or ritaphen or rubifen or 'sr 20' or tranquilyn or equasym or tsentedrin).ab,ti,kf. | 8,624 |
|  | 6. | 4 or 5 | 10,331 |
| Combined sets | | | |
|  | 7. | 3 and 6 | 5,362 |
| Limit to systematic review | | | |
|  | 8. | limit 7 to (meta analysis or "systematic review") | 155 |
|  | 9. | (systematic* adj4 (review* or overview*)).ab,ti,kf. | 208,507 |
|  | 10. | meta-analysis/ or "systematic review"/ | 205,462 |
|  | 11. | (meta-analys* or "review? of review*" or meta-synthesis or meta-ethnography or "rapid review*" or "EPPI-centre review*" or meta-review* or metareview* or "health technology assessment").ab,kf,ti. | 200,959 |
|  | 12. | 9 or 10 or 11 | 343,221 |
|  | 13. | 7 and 12 | 253 |
|  | 14. | 8 or 13 | 253 |
| Languages: (Danish or English or French or Norwegian or Swedish) | | | |
|  | 15. | 8 or 13 | 240 |
| After deduplication | | | |
|  | 16. | 8 or 13 | 238 |

**Cochrane via Wiley 2020-12-18**

|  | | | Number of publications |
| --- | --- | --- | --- |
| Attention Deficit Hyperactivity Disorder | | | |
|  | #1 | MeSH descriptor: [Attention Deficit Disorder with Hyperactivity] explode all trees | 2,750 |
|  | #2 | ("Attention Deficit Disorder with Hyperactivity" or Attention Deficit Hyperactivity Disorder* or Attention Deficit Disorder*ADHD or ADDH):ti,ab,kw | 6,419 |
|  | #3 | #1 OR #2 | 6,419 |
| Methylphenidate | | | |
|  | #4 | MeSH descriptor: [Methylphenidate] explode all trees | 1,593 |
|  | #5 | MeSH descriptor: [Dexmethylphenidate Hydrochloride] explode all trees | 40 |
|  | #6 | (dextroamphetamine OR dexamphetamine OR dextroamfetamine OR dexamfetamine OR dexmethylphenydate  OR adhansia OR aptensio OR attenta  OR centedrin OR concerta OR cotempla OR daytrana  OR difumenil OR methylphenidate OR jornay OR medanef OR medicebran OR medikinet OR mefinad OR metadate OR “methyl 2 phenyl 2 piperid 2 ylacetate” OR “methyl phenidate” OR methylfenidaat OR methylfenidate OR methylin OR methylphenidylacetate OR methylphenindate OR methylphenydate OR methypatch OR penid OR “phenidyl hydrochloride” OR phenidylate OR quasym OR quillichew OR quillivant OR rilatine OR ritalin OR  “ritalin sr” OR ritalina OR ritaline OR ritaphen OR rubifen OR “sr 20” OR tranquilyn OR equasym OR tsentedrin):ti,ab,kw | 3,677 |
|  | #7 | #4 OR #5 OR #6 | 3,688 |
| Combined sets | | | |
|  | #8 | #3 and #7 | 2,059 |
| Limit to systematic reviews | | | |
|  | #9 | 13 systematic reviews och 2 protocol | 15 |
| After deduplication | | | |
|  | #10 | #3 and #7 | 3 |

**Embase via embase.com 2020-10-21**

|  | | | Number of publications |
| --- | --- | --- | --- |
| Attention Deficit Hyperactivity Disorder | | | |
|  | 1. | 'attention deficit disorder'/de | 64,332 |
|  | 2. | adhd:ab,kw,ti OR 'attention deficit disorder*':ab,kw,ti OR 'attention deficit disorder with hyperactivity':ab,kw,ti OR 'attention deficit hyperactivity disorder*':ab,kw,ti OR addh:ab,kw,ti | 48,263 |
|  | 3. | 1 OR 2 | 69,434 |
| Methylphenidate | | | |
|  | 4. | 'methylphenidate'/de OR 'dexmethylphenidate'/de | 22,527 |
|  | 5. | adhansia:ab,kw,ti OR 'alpha phenyl 2 piperidineacetic acid methyl ester':ab,kw,ti OR 'alpha phenyl alpha acetic acid methyl ester':ab,kw,ti OR aptensio:ab,kw,ti OR attenta:ab,kw,ti OR 'c 4311-b':ab,kw,ti OR 'c4311-b':ab,kw,ti OR 'c4311b':ab,kw,ti OR centedrin:ab,kw,ti OR concerta:ab,kw,ti OR cotempla:ab,kw,ti OR 'd erythro methyl phenidylacetate':ab,kw,ti OR daytrana:ab,kw,ti OR difumenil:ab,kw,ti OR 'dl erythro methyl phenidylacetate':ab,kw,ti OR methylphenidate:ab,kw,ti OR jornay:ab,kw,ti OR medanef:ab,kw,ti OR medicebran:ab,kw,ti OR medikinet:ab,kw,ti OR mefinad:ab,kw,ti OR metadate:ab,kw,ti OR 'methyl 2 phenyl 2 piperid 2 ylacetate':ab,kw,ti OR 'methyl phenidate':ab,kw,ti OR methylfenidaat:ab,kw,ti OR methylfenidate:ab,kw,ti OR methylin:ab,kw,ti OR methylphenidylacetate:ab,kw,ti OR methylphenindate:ab,kw,ti OR methylphenydate:ab,kw,ti OR methypatch:ab,kw,ti OR penid:ab,kw,ti OR 'phenidyl hydrochloride':ab,kw,ti OR phenidylate:ab,kw,ti OR quasym:ab,kw,ti OR quillichew:ab,kw,ti OR quillivant:ab,kw,ti OR rilatine:ab,kw,ti OR ritalin:ab,kw,ti OR 'ritalin sr':ab,kw,ti OR ritalina:ab,kw,ti OR ritaline:ab,kw,ti OR ritaphen:ab,kw,ti OR rubifen:ab,kw,ti OR 'sr 20':ab,kw,ti OR tranquilyn:ab,kw,ti OR equasym:ab,kw,ti OR tsentedrin:ab,kw,ti OR dexmethylphenidate:ab,kw,ti OR dextroamphetamine:ab,kw,ti OR dexamphetamine:ab,kw,ti OR dextroamfetamine:ab,kw,ti OR dexamfetamine:ab,kw,ti | 11,987 |
|  | 6. | 4 OR 5 | 24,400 |
| Combined sets | | | |
|  | 7. | 3 AND 6 | 11,328 |
| Limit to systematic review | | | |
|  | 8. | 3 AND 6 AND ([cochrane review]/lim OR [systematic review]/lim OR [meta analysis]/lim) | 521 |
|  | 9. | (systematic* NEAR/4 (review* OR overview*)):ab,kw,ti | 258,839 |
|  | 10. | 'systematic review'/de OR 'meta analysis'/de | 373,155 |
|  | 11. | 'meta analys*':ab,kw,ti OR 'review$ of review*':ab,kw,ti OR 'meta synthesis':ab,kw,ti OR 'meta ethnography':ab,kw,ti OR 'rapid review*':ab,kw,ti OR 'eppi-centre review*':ab,kw,ti OR 'meta review*':ab,kw,ti OR metareview*:ab,kw,ti OR 'health technology assessment':ab,kw,ti | 249,784 |
|  | 12. | 9 OR 10 OR 11 | 491,356 |
|  | 13. | 7 AND 12 | 624 |
|  | 14. | 8 OR 13 | 632 |
| Languages: (Danish or English or French or Norwegian or Swedish) | | | |
|  | 15. | 8 OR 13 | 604 |
| After deduplication | | | |
|  | 16. | 8 OR 13 | 386 |

**PsycINFO via EBSCOhost 2020-12-18**

|  | | | Number of publications |
| --- | --- | --- | --- |
| Attention Deficit Hyperactivity Disorder | | | |
|  | 1. | (DE "Attention Deficit Disorder" OR DE "Attention Deficit Disorder with Hyperactivity") | 31,630 |
|  | 2. | TI (adhd OR 'attention deficit disorder*' OR 'attention deficit disorder with hyperactivity' OR 'attention deficit hyperactivity disorder' OR addh) OR AB (adhd OR 'attention deficit disorder*' OR 'attention deficit disorder with hyperactivity' OR 'attention deficit hyperactivity disorder' OR addh) OR SU (adhd OR 'attention deficit disorder*' OR 'attention deficit disorder with hyperactivity' OR 'attention deficit hyperactivity disorder' OR addh) | 43,974 |
|  | 3. | 1 OR 2 | 43,974 |
| Methylphenidate | | | |
|  | 4. | (DE "Methylphenidate") OR (DE "Dextroamphetamine") | 8,116 |
|  | 5. | TI ( adhansia OR 'alpha phenyl 2 piperidineacetic acid methyl ester' OR 'alpha phenyl alpha acetic acid methyl ester' OR aptensio OR attenta OR 'c 4311-b' OR 'c4311-b' OR 'c4311b' OR centedrin OR concerta OR cotempla OR 'd erythro methyl phenidylacetate' OR daytrana OR difumenil OR 'dl erythro methyl phenidylacetate' OR methylphenidate OR jornay OR medanef OR medicebran OR medikinet OR mefinad OR metadate OR 'methyl 2 phenyl 2 piperid 2 ylacetate' OR 'methyl phenidate' OR methylfenidaat OR methylfenidate OR methylin OR methylphenidylacetate OR methylphenindate OR methylphenydate OR methypatch OR penid OR 'phenidyl hydrochloride' OR phenidylate OR quasym OR quillichew OR quillivant OR rilatine OR ritalin OR 'ritalin sr' OR ritalina OR ritaline OR ritaphen OR rubifen OR 'sr 20' OR tranquilyn OR equasym OR tsentedrin OR dexmethylphenidate OR dextroamphetamine OR dexamphetamine OR dextroamfetamine OR dexamfetamine ) OR AB ( adhansia OR 'alpha phenyl 2 piperidineacetic acid methyl ester' OR 'alpha phenyl alpha acetic acid methyl ester' OR aptensio OR attenta OR 'c 4311-b' OR 'c4311-b' OR 'c4311b' OR centedrin OR concerta OR cotempla OR 'd erythro methyl phenidylacetate' OR daytrana OR difumenil OR 'dl erythro methyl phenidylacetate' OR methylphenidate OR jornay OR medanef OR medicebran OR medikinet OR mefinad OR metadate OR 'methyl 2 phenyl 2 piperid 2 ylacetate' OR 'methyl phenidate' OR methylfenidaat OR methylfenidate OR methylin OR methylphenidylacetate OR methylphenindate OR methylphenydate OR methypatch OR penid OR 'phenidyl hydrochloride' OR phenidylate OR quasym OR quillichew OR quillivant OR rilatine OR ritalin OR 'ritalin sr' OR ritalina OR ritaline OR ritaphen OR rubifen OR 'sr 20' OR tranquilyn OR equasym OR tsentedrin OR dexmethylphenidate OR dextroamphetamine OR dexamphetamine OR dextroamfetamine OR dexamfetamine ) OR SU(( adhansia OR 'alpha phenyl 2 piperidineacetic acid methyl ester' OR 'alpha phenyl alpha acetic acid methyl ester' OR aptensio OR attenta OR 'c 4311-b' OR 'c4311-b' OR 'c4311b' OR centedrin OR concerta OR cotempla OR 'd erythro methyl phenidylacetate' OR daytrana OR difumenil OR 'dl erythro methyl phenidylacetate' OR methylphenidate OR jornay OR medanef OR medicebran OR medikinet OR mefinad OR metadate OR 'methyl 2 phenyl 2 piperid 2 ylacetate' OR 'methyl phenidate' OR methylfenidaat OR methylfenidate OR methylin OR methylphenidylacetate OR methylphenindate OR methylphenydate OR methypatch OR penid OR 'phenidyl hydrochloride' OR phenidylate OR quasym OR quillichew OR quillivant OR rilatine OR ritalin OR 'ritalin sr' OR ritalina OR ritaline OR ritaphen OR rubifen OR 'sr 20' OR tranquilyn OR equasym OR tsentedrin OR dexmethylphenidate OR dextroamphetamine OR dexamphetamine OR dextroamfetamine OR dexamfetamine ) | 9,716 |
|  | 6. | 4 OR 5 | 9,716 |
| Combined Sets | | | |
|  | 7. | 3 AND 6 | 4,009 |
| Limit to systematic review | | | |
|  | 8. | 3 AND 6  Limiters - Methodology: -Systematic Review, META ANALYSIS, METASYNTHESIS; Exclude Dissertations | 109 |
|  | 9. | TI (systematic* N3 review*) OR AB (systematic* N3 overview*) OR SU (systematic* N3 overview*) | 24,125 |
|  | 10. | TI ( review* N3 (meta or quality or integrative) ) OR AB ( review* N3 (meta or quality or integrative) OR SU ( review* N3 (meta or quality or integrative) ) | 17,381 |
|  | 11. | TI evidence AND TI ( review* or meta-analys* ) | 3,741 |
|  | 12. | TI ( meta-analys* or " review* of review*" or meta-synthesis or meta-ethnography or "rapid review*" or "EPPI-centre review*" or meta-review* or metareview* or "health technology assessment" ) OR AB ( meta-analys* or " review* of review*" or meta-synthesis or meta-ethnography or "rapid review*" or "EPPI-centre review*" or meta-review* or metareview* or "health technology assessment" ) OR SU ( meta-analys* or " review* of review*" or meta-synthesis or meta-ethnography or "rapid review*" or "EPPI-centre review*" or meta-review* or metareview* or "health technology assessment" ) | 40,109 |
|  | 13. | 9 OR 10 OR 11 OR 12 | 63,432 |
|  | 14. | 7 AND 13 | 142 |
|  | 15. | DE "Meta Analysis" OR DE "Systematic Review" | 5,295 |
|  | 16. | 7 AND 15 | 6 |
|  | 17. | 8 OR 14 OR 16 | 162 |
| Languages (Danish or English or French or Norwegian or Swedish) | | | |
|  | 18. | 8 OR 14 OR 16 | 155 |
| After deduplication | | | |
|  | 19. | 8 OR 14 OR 16 | 38 |
