# Supplementary material for: Conflict of interest and risk of bias in systematic reviews on methylphenidate for attention-deficit hyperactivity disorder: a cross-sectional study
Source: Syst Rev. 2023 Sep 26;12:175. doi: 10.1186/s13643-023-02342-x (PMC10521496; doi:10.1186/s13643-023-02342-x)
Supplement: Supplementary file 2 — Additional file 2. Excluded publications. [file 13643_2023_2342_MOESM2_ESM.docx]

Additional file 2. Excluded publications (n=112)

| Publication | Reason for exclusion |
| --- | --- |
| 1. Auffret M, Nourredine M, Cottin J, Cucherat M. (2019). Methylphenidate during pregnancy and the risk of congenital major malformations: A systematic review and meta-analysis. *Fundamental and Clinical Pharmacology, 33*, 27. | Wrong publication: conference abstract |
| 1. Bagot KS, Kaminer Y. (2014). Efficacy of stimulants for cognitive enhancement in non-attention deficit hyperactivity disorder youth: a systematic review. *Addiction, 109*(4), 547-557. | Wrong population:  not ADHD |
| 1. Benkert D, Krause KH, Wasem J, Aidelsburger P. Effectiveness of pharmaceutical therapy of ADHD (Attention-Deficit/Hyperactivity Disorder) in adults - health technology assessment. GMS Health Technol Assess. 2010 Sep 7;6:Doc13. | No data:  no separate analysis of MPH |
| 1. Bitter I, Angyalosi A, Czobor P. (2012). Pharmacological treatment of adult ADHD. *Current Opinion in Psychiatry, 25*(6), 529-534. | Wrong publication:  not a SR |
| 1. Bolea-Alamanac BM, Green A, Verma G, Maxwell P, Davies SJC. Methylphenidate use in pregnancy and lactation: a systematic review of evidence. British journal of clinical pharmacology. 2014;77(1):96-101. | Wrong focus:  not comparing MPH to other treatments |
| 1. Bui A, Joseph A, Sam D, Lane C, Madireddy S, Afghani B. (2018). Cardiovascular safety of methylphenidate (ritalin) in treating paediatric patients with ADHD (attention deficit hyperactivity disorder): A literature review. *Journal of Investigative Medicine, 66*(1), 68-69. | Wrong publication:  abstract |
| 1. Carucci S, Balia C, Gagliano A, Lampis A, Buitelaar JK, Danckaerts M, Dittmann RW, Garas P, Hollis C, Inglis S, Konrad K, Kovshoff H, Liddle EB, McCarthy S, Nagy P, Panei P, Romaniello R, Usala T, Wong ICK, Banaschewski T, Sonuga-Barke E, Coghill D, Zuddas A; ADDUCE Consortium. Long term methylphenidate exposure and growth in children and adolescents with ADHD. A systematic review and meta-analysis. Neurosci Biobehav Rev. 2021 Jan;120:509-525. | Wrong focus:  not comparing MPH to other treatments |
| 1. Carucci S, Usala T, Granitzio F, Balia C, Coghill D, Zuddas A. (2013). Growth on stimulant medication: Effects in children with ADHD. *European Neuropsychopharmacology, 23*, S605. | Wrong publication:  abstract |
| 1. Castells X, Ramon M, Cunill R, Olivé C, Serrano D. Relationship Between Treatment Duration and Efficacy of Pharmacological Treatment for ADHD: A Meta-Analysis and Meta-Regression of 87 Randomized Controlled Clinical Trials. J Atten Disord. 2021 Aug;25(10):1352-1361. | Wrong focus:  not comparing MPH to other treatments |
| 1. Castells X, Ramos-Quiroga JA, Rigau D, Bosch R, Nogueira M, Vidal X, Casas M. (2011). Efficacy of methylphenidate for adults with attention-deficit hyperactivity disorder: a meta-regression analysis. *CNS Drugs, 25*(2), 157-169. | Wrong version:  an update included |
| 1. Cerrillo-Urbina AJ, Garcia-Hermoso A, Pardo-Guijarro MJ, Sanchez-Lopez M, Santos-Gomez JL, Martinez-Vizcaino V. (2018). The Effects of Long-Acting Stimulant and Nonstimulant Medications in Children and Adolescents with Attention-Deficit/Hyperactivity Disorder: A Meta-Analysis of Randomized Controlled Trials. *Journal of Child and Adolescent Psychopharmacology, 28*(8), 494-507. | No data:  no separate analysis of MPH |
| 1. Charach A, Carson P, Fox S, Ali MU, Beckett J, Lim CG. (2013). Interventions for preschool children at high risk for ADHD: a comparative effectiveness review. *Pediatrics, 131*(5), e1584-1604. | Wrong population:  not ADHD |
| 1. Charach A, Carson P, Fox S, Haq M, & Lim C. (2012). Comparing interventions for preschoolers with ADHD or disruptive behavior. *Neuropsychiatrie de l'Enfance et de l'Adolescence, 60*(5), S91-S92. | Wrong population:  not only ADHD |
| 1. Charach A, Dashti B, Carson P, Booker L, Lim CG, Lillie E, Yeung E, Ma J, Raina P, Schachar R. Attention Deficit Hyperactivity Disorder: Effectiveness of Treatment in At-Risk Preschoolers; Long-Term Effectiveness in All Ages; and Variability in Prevalence, Diagnosis, and Treatment [Internet]. Rockville (MD): Agency for Healthcare Research and Quality (US); 2011 Oct. Report No.: 12-EHC003-EF. | Wrong population:  not only ADHD |
| 1. Ching C, Eslick GD, Poulton AS. (2019). Evaluation of Methylphenidate Safety and Maximum-Dose Titration Rationale in Attention-Deficit/Hyperactivity Disorder: A Meta-analysis. *JAMA Pediatrics, 173*(7), 630-639. | Wrong focus:  not comparing MPH to other treatments |
| 1. Ching C, Poulton A, Eslick G. (2017). Evaluating the safety of methylphenidate and rationale for a maximum dose for titration: A meta-analysis of randomised controlled trials and case studies. *Journal of Paediatrics and Child Health, 53*, 19. | Wrong focus:  not comparing MPH to other treatments |
| 1. Cohen SC, Mulqueen JM, Ferracioli-Oda E, Stuckelman ZD, Coughlin CG, Leckman JF, Bloch MH. (2015). Meta-Analysis: Risk of Tics Associated With Psychostimulant Use in Randomized, Placebo-Controlled Trials. *Journal of the American Academy of Child and Adolescent Psychiatry, 54*(9), 728-736. | Wrong publication:  not a SR |
| 1. Conners CK. (2002). Forty years of methylphenidate treatment in Attention-Deficit/ Hyperactivity Disorder. *Journal of Attention Disorders, 6*, S17-30. | Wrong publication:  not a SR |
| 1. Cook J, Lloyd-Jones M, Arunogiri S, Ogden E, Bonomo Y. (2017). Managing attention deficit hyperactivity disorder in adults using illicit psychostimulants: A systematic review. *Australian and New Zealand Journal of Psychiatry, 51*(9), 876-885. | No data:  no separate analysis of MPH |
| 1. Cortese S, D’Acunto G, Konofal E, Masi G, Vitiello B. (2017). New Formulations of Methylphenidate for the Treatment of Attention-Deficit/Hyperactivity Disorder: Pharmacokinetics, Efficacy, and Tolerability. *CNS Drugs, 31*(2), 149-160. | Wrong publication:  not a SR |
| 1. Cunill R, Castells X, Capellà D. (2013). The effect of treatment duration on methylphenidate discontinuation in adults with ADHD: A meta-analysis. *European Neuropsychopharmacology, 23*, S607. | No data:  no separate analysis of MPH |
| 1. Cunill R, Castells X, Tobias A, Capellà D. (2016). Efficacy, safety and variability in pharmacotherapy for adults with attention deficit hyperactivity disorder: A meta-analysis and meta-regression in over 9000 patients. *Psychopharmacology, 233*(2), 187-197. | No data:  no separate analysis of MPH |
| 1. Donnelly M, Haby MM, Carter R, Andrews G, Vos T. (2004). Cost-effectiveness of dexamphetamine and methylphenidate for the treatment of childhood attention deficit hyperactivity disorder. *The Australian and New Zealand journal of psychiatry, 38*(8), 592-601. | Wrong publication:  not a SR |
| 1. Epstein T, Patsopoulos NA, Weiser, M. (2009). Methylphenidate for adults with attention deficit-hyperactivity disorder, a systematic review. *European Neuropsychopharmacology, 19*, S341. | Wrong publication:  abstract |
| 1. Epstein T, Patsopoulos NA, Weiser M. WITHDRAWN: Immediate-release methylphenidate for attention deficit hyperactivity disorder (ADHD) in adults. Cochrane Database Syst Rev. 2016 May 26;(5):CD005041. doi: 10.1002/14651858.CD005041.pub3. | Withdrawn |
| 1. Epstein T, Patsopoulos NA, Weiser M. (2016). WITHDRAWN: Immediate-release methylphenidate for attention deficit hyperactivity disorder (ADHD) in adults. *The Cochrane database of systematic reviews*(5), CD005041. | Withdrawn |
| 1. Faraone SV. Using Meta-analysis to Compare the Efficacy of Medications for Attention-Deficit/Hyperactivity Disorder in Youths. P T. 2009 Dec;34(12):678-94. | Wrong publication:  not a SR |
| 1. Faraone SV, Biederman J, Roe C. (2002). Comparative efficacy of Adderall and methylphenidate in attention-deficit/hyperactivity disorder: a meta-analysis. *Journal of Clinical Psychopharmacology, 22*(5), 468-473. | Wrong publication:  not a SR |
| 1. Faraone SV, Biederman J, Spencer TJ, Aleardi M. (2006). Comparing the efficacy of medications for ADHD using meta-analysis. *MedGenMed : Medscape general medicine, 8*(4), 4. | Wrong publication:  not a SR |
| 1. Faraone SV, Buitelaar J. (2010). Comparing the efficacy of stimulants for ADHD in children and adolescents using meta-analysis. *European Child & Adolescent Psychiatry, 19*(4), 353-364. | Wrong publication:  not a SR |
| 1. Faraone SV, Glatt SJ. (2010). A comparison of the efficacy of medications for adult attention-deficit/ hyperactivity disorder using meta-analysis of effect sizes. *Journal of Clinical Psychiatry, 71*(6), 754-763. | Wrong publication:  not a SR |
| 1. Faraone SV, Po MD, Komolova M, Cortese S. (2018). Impact of the Placebo Rate on the Relative Risk of Sleep-Related Adverse Events Reported in Studies of Methylphenidate in Youth With ADHD: A Meta-Analysis. *Journal of the American Academy of Child and Adolescent Psychiatry, 57*(10), S170. | Wrong publication:  not a SR |
| 1. Faraone SV, Spencer T, Aleardi M, Pagano C, Biederman J. (2004). Meta-analysis of the efficacy of methylphenidate for treating adult attention-deficit/hyperactivity disorder. *Journal of Clinical Psychopharmacology, 24*(1), 24-29. | Wrong publication:  not a SR |
| 1. Faria JCM, Ferreira LA, Duarte LJR, Anicio VTS, De Pádua CAM, Perini E. (2018). “Real-world” effectiveness of methylphenidate in improving academic achievement of attention deficit hyperactivity disorder (ADHD) students-A systematic review. *Pharmacoepidemiology and Drug Safety, 27*, 99-100. | Wrong publication:  abstract |
| 1. Fredriksen M, Halmoy A, Faraone SV, Haavik J. (2013). Long-term efficacy and safety of treatment with stimulants and atomoxetine in adult ADHD: a review of controlled and naturalistic studies. *European neuropsychopharmacology : the journal of the European College of Neuropsychopharmacology, 23*(6), 508-527. | Wrong focus:  not comparing MPH to other treatments |
| 1. Fredriksen M, Peleikis DE. (2016). Long-Term Pharmacotherapy of Adults With Attention Deficit Hyperactivity Disorder: A Literature Review and Clinical Study. *Basic & clinical pharmacology & toxicology, 118*(1), 23-31. | No data:  no separate analysis of MPH |
| 1. Gadow KD, Sverd J. (2006). Attention deficit hyperactivity disorder, chronic tic disorder, and methylphenidate. *Advances in neurology, 99*, 197-207. | Wrong publication  not a SR |
| 1. Ghanbari M, Ghanizadeh A. (2014). Methylphenidate improves handwriting of children with ADHD; a systematic review of controlled clinical trials. *International Journal of Neuropsychopharmacology, 17*, 122. | Wrong publication:  abstract |
| 1. Gobbo MA, Louza MR. (2014). Influence of stimulant and non-stimulant drug treatment on driving performance in patients with attention deficit hyperactivity disorder: a systematic review. *European neuropsychopharmacology : the journal of the European College of Neuropsychopharmacology, 24*(9), 1425-1443. | No data:  no separate analysis of MPH or comparison |
| 1. Gurgel W, Aoyama R, Polanczyk G. (2019). Pharmacological interventions for ADHD in preschool children: A systematic review. *ADHD Attention Deficit and Hyperactivity Disorders, 11*(1), S64-S65. | Wrong publication:  abstract |
| 1. Hazell PL, Kohn MR, Dickson R., Walton RJ, Grange RE, Wyk GW. (2011). Core ADHD symptom improvement with atomoxetine versus methylphenidate: a direct comparison meta-analysis. *Journal of Attention Disorders, 15*(8), 674-683. | Wrong publication:  not a SR |
| 1. Hennissen L, Bakker M, Buitelaar JK. (2015). Cardiovascular effects of methylphenidate, amphetamines and atomoxetine in children and adolescents with attention deficit hyperactivity disorder. *European Child and Adolescent Psychiatry, 24*(1), S50. | Wrong publication:  not a SR |
| 1. Hodgkins P, Shaw M, McCarthy S, Sallee FR. (2012). The pharmacology and clinical outcomes of amphetamines to treat ADHD: does composition matter? *CNS Drugs, 26*(3), 245-268. | Wrong publication:  not a SR |
| 1. Humphreys, KL, Eng T, Lee SS. (2013). Stimulant medication and substance use outcomes ameta-analysis. *JAMA Psychiatry, 70*(7), 740-749. | No data:  no separate analysis of MPH |
| 1. Jadad AR, Booker L, Gauld M, Kakuma R, Boyle M, Cunningham CE, Kim M, Schachar R. The treatment of attention-deficit hyperactivity disorder: an annotated bibliography and critical appraisal of published systematic reviews and metaanalyses. Can J Psychiatry. 1999 Dec;44(10):1025-35. | Wrong publication:  not a SR |
| 1. Jadad AR, Boyle M, Cunningham C, Kim M, Schachar R. (1999). Treatment of attention-deficit/hyperactivity disorder. *Evidence report/technology assessment (Summary)* (11), i-341. | Wrong publication:  not a SR |
| 1. Jerome L, Habinski L. (2006). Attention-deficit/hyperactivity disorder (ADHD) and driving risk: A review of the literature and a methodological critique. *Current Psychiatry Reports, 8*(5), 416-426. | Wrong publication:  not a SR |
| 1. Jiang HY, Zhang X, Jiang CM, Fu HB. (2019). Maternal and neonatal outcomes after exposure to ADHD medication during pregnancy: A systematic review and meta-analysis. *Pharmacoepidemiology and Drug Safety, 28*(3), 288-295. | Wrong intervention:  not MPH |
| 1. Joseph A, Ayyagari R, Bischof M, Cai S, Xi, M, Zhanabekova Z, Sikirica V. (2014). Systematic literature review and mixed treatment comparison of GXR versus other treatments in children and adolescents with attention deficit hyperactivity disorder (ADHD). *Value in Health, 17*(7), A454. | Wrong publication:  abstract |
| 1. Joseph A, Xie J, Bischof M, Zhanabekova Z., Cai S, Zhuo Y, Sikirica V. (2015). Systematic literature review and mixed treatment comparison of guanfacine extended release (GXR) versus other pharmacotherapies in children and adolescents with attention deficit/hyperactivity disorder (ADHD). *European Child and Adolescent Psychiatry, 24*(1), S166-S167. | Wrong publication:  abstract |
| 1. Kavale K. (1982). The efficacy of stimulant drug treatment for hyperactivity: a meta-analysis. *Journal of learning disabilities, 15*(5), 280-289. | Wrong publication:  Not a SR |
| 1. Keen D, Hadijikoumi I. (2008). ADHD in children and adolescents. *BMJ clinical evidence, 2008*. | Wrong version:  an update included |
| 1. King S, Griffin S, Hodges Z, Weatherly H, Asseburg C, Richardson G, Golder S, Taylor E, Drummond M, Riemsma R. A systematic review and economic model of the effectiveness and cost-effectiveness of methylphenidate, dexamfetamine and atomoxetine for the treatment of attention deficit hyperactivity disorder in children and adolescents. Health Technol Assess. 2006 Jul;10(23):iii-iv, xiii-146. | No data: no separate analysis of MPH |
| 1. Klassen A, Miller A, Raina P, Lee SK, Olsen L. (1999). Attention-deficit hyperactivity disorder in children and youth: A quantitative systematic review of the efficacy of different management strategies. *Canadian Journal of Psychiatry, 44*(10), 1007-1016. | No data:  no separate analysis of MPH |
| 1. Kok FM, Groen Y, Fuermaier ABM, Tucha O. (2020). The female side of pharmacotherapy for ADHD-A systematic literature review. *PLoS ONE, 15*(9), e0239257. | Wrong focus:  not comparing MPH to other treatments |
| 1. Krinzinger H, Hall CL, Groom MJ, Ansari MT, Banaschewski T, Buitelaar JK, Liddle EB. (2019). Neurological and psychiatric adverse effects of long-term methylphenidate treatment in ADHD: A map of the current evidence. *Neuroscience and Biobehavioral Reviews, 107*, 945-968. | Wrong publication:  Not a SR |
| 1. Krogh HB, Storebø OJ, Faltinsen E, et al Methodological advantages and disadvantages of parallel and crossover randomised clinical trials on methylphenidate for attention deficit hyperactivity disorder: a systematic review and meta-analysesBMJ Open 2019;9:e026478. | Wrong focus:  comparison of study designs |
| 1. Kösters M, Weinmann S, Becker T. (2010). A meta-analysis of the effectiveness of methylphenidate in the treatment of adult ADHD. *European Psychiatry, 25*. | Wrong publication:  abstract |
| 1. Koesters M, Becker T, Kilian R, Fegert JM, Weinmann S. Limits of meta-analysis: methylphenidate in the treatment of adult attention-deficit hyperactivity disorder. Journal of psychopharmacology (Oxford, England). 2009;23(7):733-44. | Wrong focus:  Evaluates methodology |
| 1. Leite BF, Vidal JS, Silva AS, Brito GV, Souza KM, Freitas MG, Silveira DS. (2015). Evidence of efficacy and safety of methylphenidate in the treatment of children or adolescents with attention deficit disorder and hyperactivity (ADHD). *Value in Health, 18*(7), A837. | Wrong publication: conference abstract |
| 1. Levy, F. (1993). Side effects of stimulant use. *Journal of Paediatrics and Child Health, 29*(4), 250-254. | Wrong publication:  Not a SR |
| 1. Li L, Sujan AC, Butwicka A, Chang Z, Cortese S, Quinn P, Viktorin A, Öberg AS, D'Onofrio BM, Larsson H. Associations of Prescribed ADHD Medication in Pregnancy with Pregnancy-Related and Offspring Outcomes: A Systematic Review. CNS Drugs. 2020 Jul;34(7):731-747. doi: 10.1007/s40263-020-00728-2. | Wrong focus:  not comparing MPH to other treatments |
| 1. Lie N. (1998). Methylphenidate/treatment of adults with AD/HD: Does it help? *Tidsskrift for den Norske Laegeforening, 118*(27), 4223-4227. | Wrong publication:  Not a SR |
| 1. Locatelli I, Venišnik K. (2016). A network meta-analysis of atomoxetine, methylphenidate, lisdexamfetamine, and bupropion for the treatment of attention deficit hyperactivity disorder in children and adolescents. *Value in Health, 19*(7), A521-A522. | Wrong publication: Conference abstract |
| 1. Losier BJ, McGrath PJ, Klein RM. (1996). Error patterns on the continuous performance test in non-medicated and medicated samples of children with and without ADHD: a meta-analytic review. *Journal of child psychology and psychiatry, and allied disciplines, 37*(8), 971-987. | Wrong focus:  not comparing MPH to other treatments |
| 1. Luan R, Mu Z, Yue F, He S. (2017). Efficacy and Tolerability of Different Interventions in Children and Adolescents with Attention Deficit Hyperactivity Disorder. *Frontiers in psychiatry, 8*, 229. | Wrong publication:  Not a SR |
| 1. Maia CRM, Cortese S, Caye A, Deakin TK, Polanczyk GV, Polanczyk CA, Rohde LAP. (2017). Long-Term Efficacy of Methylphenidate Immediate-Release for the Treatment of Childhood ADHD. *Journal of Attention Disorders, 21*(1), 3-13. | Wrong focus:  not comparing MPH to other treatments |
| 1. Man KKC, Ip P, Chan EW, Law S-L, Leung MTY, Ma EXY, Quek W-T. (2017). Effectiveness of Pharmacological Treatment for Attention-Deficit/Hyperactivity Disorder on Physical Injuries: A Systematic Review and Meta-Analysis of Observational Studies. CNS Drugs , 31 (12) pp. 1043-1055. | Wrong focus:  not comparing MPH to other treatments |
| 1. Maneeton N, Maneeton B, Woottiluk P, Suttajit S, Charnsil C, Srisurapanont M. (2015). A systematic review of dexmethylphenidate versus placebo in child and adolescent ADHD: A meta-analysis of randomized, controlled trials. *European Neuropsychopharmacology, 25*, S642. | Wrong intervention:  not MPH |
| 1. Maneeton N, Maneeton B, Woottiluk P, Suttaji, S, Likhitsathian S, Charnsil C, Srisurapanont M. (2015). Comparative efficacy, acceptability, and tolerability of dexmethylphenidate versus placebo in child and adolescent ADHD: a meta-analysis of randomized controlled trials. *Neuropsychiatric Disease and Treatment, 11*, 2943-2952. | Wrong publication:  Not a SR |
| 1. McDonagh MS, Peterson K, Thakurta S, Low A. Drug Class Review: Pharmacologic Treatments for Attention Deficit Hyperactivity Disorder: Final Update 4 Report. Oregon Health & Science University, Portland (OR); 2011. | Wrong publication:  Not a SR |
| 1. Mészáros Á, Czobor P, Bálint S, Komlósi S, Simon V, Bitter I. (2009). Pharmacotherapy of adult attention deficit hyperactivity disorder (ADHD): A meta-analysis. *International Journal of Neuropsychopharmacology, 12*(8), 1137-1147. | Wrong publication:  Not a SR |
| 1. Mick E, McManus DD, Goldberg RJ. (2013). Meta-analysis of increased heart rate and blood pressure associated with CNS stimulant treatment of ADHD in adults. *European Neuropsychopharmacology, 23*(6), 534-541. | Wrong focus:  not comparing MPH to other treatments |
| 1. Miller J, Perera B, Shankar R. (2020). Clinical guidance on pharmacotherapy for the treatment of attention-deficit hyperactivity disorder (ADHD) for people with intellectual disability. *Expert Opinion on Pharmacotherapy*, 1-17. | Wrong publication:  Not a SR |
| 1. Millichap JG. (2015). Risk of Tics with Psychostimulants for ADHD. *Pediatric neurology briefs, 29*(12), 95. | Wrong publication:  Not a SR |
| 1. Moukhtarian TR, Cooper RE, Vassos E, Moran P, Asherson P. (2017). Effects of stimulants and atomoxetine on emotional lability in adults: A systematic review and meta-analysis. *European psychiatry : the journal of the Association of European Psychiatrists, 44*, 198-207. | Wrong focus:  not comparing MPH to other treatments |
| 1. Myer N, Bol J, Faraone S, Krause D. (2019). PHARMACOGENETICS PREDICTORS OF METHYLPHENIDATE EFFICACY RESPONSE IN CHILDHOOD ADHD. *European Neuropsychopharmacology, 29*, S957-S958. | Wrong publication:  abstract |
| 1. Nolan M, Carr A. (2000). Attention deficit hyperactivity disorder. In Carr, A. (ed) What works with children and adolescents? A Critical review of Psychological interventions with Children, Adolescents and their Familes (pp. 65-101).   London: Routledge. | Wrong publication:  Not a SR |
| 1. Palumbo D, Spencer T, Lynch J, Co-Chien H, Faraone SV. (2004). Emergence of tics in children with ADHD: Impact of once-daily OROS® methylphenidate therapy. *Journal of Child and Adolescent Psychopharmacology, 14*(2), 185-194. | Wrong publication:  Not a SR |
| 1. Peterson K, McDonagh MS, Fu R. (2008). Comparative benefits and harms of competing medications for adults with attention-deficit hyperactivity disorder: a systematic review and indirect comparison meta-analysis. *Psychopharmacology, 197*(1), 1-11. | No data:  no separate analysis of MPH |
| 1. Pringsheim T, Steeves T. (2011). Pharmacological treatment for Attention Deficit Hyperactivity Disorder (ADHD) in children with comorbid tic disorders. *The Cochrane database of systematic reviews*(4), CD007990. | No data:  no separate analysis of MPH |
| 1. Ravi M, Ickowicz A. (2015). Epilepsy, Attention-Deficit/Hyperactivity Disorder and methylphenidate: Critical examination of guiding evidence. *Journal of the Canadian Academy of Child and Adolescent Psychiatry, 25*(1), 50-58. | Wrong publication:  Not a SR |
| 1. Ren J, Li Y, Yue S, Yue J, Li Q, Lv M, Tie L. (2017). The comparison of methylphenidate and atomoxetine on cognitive functions in children and adolescents with ADHD: A meta-analysis. *ADHD Attention Deficit and Hyperactivity Disorders, 9*(1), S40. | Wrong publication:  abstract |
| 1. Riera M, Castells X, Tobias A, Cunill R, Blanco L, Capella D. (2017). Discontinuation of pharmacological treatment of children and adolescents with attention deficit hyperactivity disorder: meta-analysis of 63 studies enrolling 11,788 patients. *Psychopharmacology, 234*(17), 2657-2671. | No data:  no separate analysis of MPH |
| 1. Santos PH, Goncalves R, Pedroso S. How does methylphenidate affect default mode network? A systematic review. Rev Neurol. 2019 May 16;68(10):417-425. | No data:  no separate analysis of MPH |
| 1. Schachar R, Jadad AR, Gauld M, Boyle M, Booker L, Snider A, Kim M, Cunningham C. Attention-deficit hyperactivity disorder: critical appraisal of extended treatment studies. Can J Psychiatry. 2002 May;47(4):337-48. | No data:  no separate analysis of MPH |
| 1. Schachter HM, Pham B, King J, Langford S, Moher D. (2001). How efficacious and safe is short-acting methylphenidate for the treatment of attention-deficit disorder in children and adolescents? A meta-analysis. *CMAJ : Canadian Medical Association journal = journal de l'Association medicale canadienne, 165*(11), 1475-1488. | Wrong population:  ADD, not ADHD |
| 1. Solmi M, Fornaro M, Ostinelli EG, Zangani C, Croatto G, Monaco F, Krinitski D, Fusar-Poli P, Correll CU. Safety of 80 antidepressants, antipsychotics, anti-attention-deficit/hyperactivity medications and mood stabilizers in children and adolescents with psychiatric disorders: a large scale systematic meta-review of 78 adverse effects. World Psychiatry. 2020 Jun;19(2):214-232. | Wrong publication:  Not a SR |
| 1. Spalding W, Toor K, Cope S, Khachatryan A, Jansen J, Nierenberg, A. (2017). Comparative efficacy and tolerability of lisdexamfetamine versus other treatments for adults with attention deficit hyperactivity disorder: A systematic literature review and network meta-analysis. *Journal of Managed Care and Specialty Pharmacy, 23*, S48-S49. | Wrong publication:  abstract |
| 1. Storebø OJ, Krogh HB, Ramstad E, Moreira-Maia CR, Holmskov M, Skoog M, Nilausen TD, Magnusson FL, Zwi M, Gillies D, Rosendal S, Groth C, Rasmussen KB, Gauci D, Kirubakaran R, Forsbøl B, Simonsen E, Gluud C. Methylphenidate for attention-deficit/hyperactivity disorder in children and adolescents: Cochrane systematic review with meta-analyses and trial sequential analyses of randomised clinical trials. BMJ. 2015 Nov 25;351:h5203. | Wrong publication:  an updated version of the SR included |
| 1. Storebø OJ, Ramstad E, Krogh HB, Nilausen TD, Skoog M, Holmskov M, Rosendal S, Groth C, Magnusson FL, Moreira-Maia CR, Gillies D, Buch Rasmussen K, Gauci D, Zwi M, Kirubakaran R, Forsbøl B, Simonsen E, Gluud C. Methylphenidate for children and adolescents with attention deficit hyperactivity disorder (ADHD). Cochrane Database Syst Rev. 2015 Nov 25;2015(11):CD009885. | Wrong publication:  an updated version of the SR included |
| 1. Storebø OJ, Pedersen N, Ramstad E, Krogh HB, Kielsholm ML, Nielsen SS, Moreira-Maia CR, Magnusson FL, Holmskov M, Nilausen TD, Skoog M, Rosendal S, Groth C, Gillies D, Rasmussen KB, Gauci D, Zwi M, Kirubakaran R, Håkonsen SJ, Aagaard L, Simonsen E, Gluud C. Methylphenidate for attention deficit hyperactivity disorder (ADHD) in children and adolescents: assessment of harmful effects in non-randomised studies.   In: Challenges to evidence-based healthcare and Cochrane. Abstracts of the 24th Cochrane Colloquium; 2016 | Wrong publication:  an updated version of the SR included |
| 1. Stuckelman ZD, Mulqueen JM, Ferracioli-Oda E, Cohen SC, Coughlin CG, Leckman JF, Bloch MH. (2017). Risk of Irritability With Psychostimulant Treatment in Children With ADHD: A Meta-Analysis. *The Journal of Clinical Psychiatry, 78*(6), e648-e655. | Wrong publication:  not a SR |
| 1. Stuhec M, Lukic P, Locatelli I. (2016). Comparative efficacy of lisdexamfetamine, mixed amphetamine salts and methylphenidate in treatment of attention deficit hyperactivity disorder in adults: A systematic review and meta-analysis. *European Neuropsychopharmacology, 26*, S446. | Wrong publication:  abstract |
| 1. Sturman N, Deckx L, van Driel ML. (2017). Methylphenidate for children and adolescents with autism spectrum disorder. *The Cochrane database of systematic reviews,* Nov 21;11(11): CD011144 | Wrong population:  not ADHD, or ADHD symptoms |
| 1. No authors listed. Sustained-release methylphenidate. New pharmaceutical forms: A slight advantage for a small number of children. (2004). *Prescrire International, 13*(74), 203-206. | Wrong publication:  not a SR |
| 1. Swanson JM, McBurnett K, Christian DL, Wigal T. (1995). Stimulant medications and the treatment of children with ADHD. *Advances in Clinical Child Psychology, 17*, 265-322. | Wrong publication:  not a SR |
| 1. Tamminga H, Reneman L, Huizenga H, Geurts H. (2014). Effects of methylphenidate on cognition in ADHD across the lifespan: Preliminary results of a meta-regression analysis. *European Neuropsychopharmacology, 24*, S727. | Wrong publication:  abstract |
| 1. Taylor, E. (2009). Sleep and tics: Problems associated with ADHD. *Journal of the American Academy of Child & Adolescent Psychiatry, 48*(9), 877-878. | Wrong publication:  not a SR |
| 1. Thurber S, Walker CE (1983). Medication and hyperactivity: a meta-analysis. *The Journal of general psychology, 108*(1), 79-86. | No data:  no separate analysis of MPH |
| 1. Torres-Acosta N, O'Keefe JH, O'Keefe CL, Lavie CJ. (2020). Cardiovascular Effects of ADHD Therapies: JACC Review Topic of the Week. *Journal of the American College of Cardiology, 76*(7), 858-866. | Wrong publication:  not a SR |
| 1. Tsujii N, Okada T, Usami M, Kuwabara H, Fujita J, Negoro H, Kawamura M, Iida J, Saito T. (2020) Effect of Continuing and Discontinuing Medications on Quality of Life After Symptomatic Remission in Attention-Deficit/Hyperactivity Disorder: A Systematic Review and Meta-Analysis. J Clin Psychiatry.   Mar 24;81(3):19r13015. | Wrong outcome:  no separate analysis of MPH |
| 1. van Wyk GW, Hazell PL, Kohn MR, Granger RE, Walton RJ (2012). How oppositionality, inattention, and hyperactivity affect response to atomoxetine versus methylphenidate: a pooled meta-analysis. *Journal of Attention Disorders, 16*(4), 314-324. | Wrong population:  ODD, not ADHD |
| 1. Vertessen K. (2019). P.831 Methylphenidate and inhibition in children with ADHD: A meta-analyses on dosage effects. *European Neuropsychopharmacology, 29*, S553-S554. | Wrong publication:  not a SR |
| 1. Westover AN, Halm EA. (2012). Do prescription stimulants increase the risk of adverse cardiovascular events?: A systematic review. *BMC cardiovascular disorders, 12*, 41. | Wrong population:  Not only ADHD |
| 1. Weyandt LL, Oster DR, Marraccini ME, Gudmundsdottir BG, Munro BA, Zavras BM, Kuhar B. Pharmacological interventions for adolescents and adults with ADHD: stimulant and nonstimulant medications and misuse of prescription stimulants. Psychol Res Behav Manag. 2014 Sep 9;7:223-49. | No data:  no separate analysis of MPH |
| 1. Wilens TE. (2003). Drug therapy for adults with attention-deficit hyperactivity disorder. *Drugs, 63*(22), 2395-2411. | No data:  no separate analysis of MPH |
| 1. Wilens TE, Biederman J, Spencer TJ, Prince J. (1995). Pharmacotherapy of adult attention deficit/hyperactivity disorder: A review. *Journal of Clinical Psychopharmacology, 15*(4), 270-279. | No data:  no separate analysis of MPH |
| 1. Wilens TE, Faraone SV, Biederman J, Gunawardene S. (2003). Does stimulant therapy of attention-deficit/hyperactivity disorder beget later substance abuse? A meta-analytic review of the literature. *Pediatrics, 111*(1), 179-185. | No data:  no separate analysis of MPH |
| 1. Wilens TE, Spencer TJ, Biederman J. (2002). A review of the pharmacotherapy of adults with attention-deficit/hyperactivity disorder. *Journal of Attention Disorders, 5*(4), 189-202. | No data:  no separate analysis of MPH |
| 1. Wilens-Władysiuk M, Skrzekowska-Baran I. (2006). The incidence of psychoactive substance abuse or dependence in ADHD patients treated and untreated with psychostimulants - An evaluation based on a systematic review. *Psychiatria i Psychologia Kliniczna, 6*(1), 24-33. | No data:  no separate analysis of MPH |
| 1. Zerovnik S, Rozman A, Locatelli I. (2018). A Network Meta-Analysis Of Medicines Used For Attention Deficit Hyperactivity Disorder In Adults. *Value in Health, 21*, S278. | Wrong publication:  abstract |
