# Supplementary material for: Conflict of interest and risk of bias in systematic reviews on methylphenidate for attention-deficit hyperactivity disorder: a cross-sectional study
Source: Syst Rev. 2023 Sep 26;12:175. doi: 10.1186/s13643-023-02342-x (PMC10521496; doi:10.1186/s13643-023-02342-x)
Supplement: Supplementary file 3 — Additional file 3. Systematic reviews included. [file 13643_2023_2342_MOESM3_ESM.docx]

Appendix 3 Systematic reviews included in the study (n=44)

Barkla XM, McArdle PA, Newbury-Birch D. Are there any potentially dangerous pharmacological effects of combining ADHD medication with alcohol and drugs of abuse? A systematic review of the literature. BMC psychiatry. 2015;15:270.

Bellino S, Mirra M, Brignolo E, Bozzatello P, Bogetto F. Pharmacological treatment of attention deficit hyperactivity disorder (ADHD): A systematic review of recent data. Curr Psychopharmacol. 2014;3(2):93-107.

Bloch MH, Panza KE, Landeros-Weisenberger A, Leckman JF. Meta-analysis: treatment of attention-deficit/hyperactivity disorder in children with comorbid tic disorders. J Am Acad Child Adolesc Psychiatry. 2009;48(9):884-93.

Bushe C, Day K, Reed V, Karlsdotter K, Berggren L, Pitcher A, et al. A network meta-analysis of atomoxetine and osmotic release oral system methylphenidate in the treatment of attention-deficit/hyperactivity disorder in adult patients. J Psychopharmacol. 2016;30(5):444-58.

Candido RCF, Menezes de Padua CA, Golder S, Junqueira DR. Immediate-release methylphenidate for attention deficit hyperactivity disorder (ADHD) in adults. Cochrane Database Syst Rev. 2021;1(1):CD013011.

Carpentier PJ, Levin FR. Pharmacological Treatment of ADHD in Addicted Patients: What Does the Literature Tell Us? Harv Rev Psychiatry. 2017;25(2):50-64.

Castells X, Cunill R, Capella D. Treatment discontinuation with methylphenidate in adults with attention deficit hyperactivity disorder: a meta-analysis of randomized clinical trials. Eur J Clin pharmacol. 2013;69(3):347-56.

Catala-Lopez F, Hutton B, Nunez-Beltran A, Page MJ, Ridao M, Macias Saint-Gerons D, et al. The pharmacological and non-pharmacological treatment of attention deficit hyperactivity disorder in children and adolescents: A systematic review with network meta-analyses of randomised trials. PloS one. 2017;12(7):e0180355.

Chan E, Fogler JM, Hammerness PG. Treatment of Attention-Deficit/Hyperactivity Disorder in Adolescents: A Systematic Review. JAMA. 2016;315(18):1997-2008.

Coghill DR, Seth S, Pedroso S, Usala T, Currie J, Gagliano A. Effects of methylphenidate on cognitive functions in children and adolescents with attention-deficit/hyperactivity disorder: evidence from a systematic review and a meta-analysis. Biol Psychiatry. 2014;76(8):603-15.

Cortese S, Adamo N, Del Giovane C, Mohr-Jensen C, Hayes AJ, Carucci S, et al. Comparative efficacy and tolerability of medications for attention-deficit hyperactivity disorder in children, adolescents, and adults: a systematic review and network meta-analysis. Lancet Psychiatry. 2018;5(9):727-38.

Coughlin CG, Cohen SC, Mulqueen JM, Ferracioli-Oda E, Stuckelman ZD, Bloch MH. Meta-Analysis: Reduced Risk of Anxiety with Psychostimulant Treatment in Children with Attention-Deficit/Hyperactivity Disorder. J Child Adolesc Psychopharmacol. 2015;25(8):611-7.

Elliott J, Johnston A, Husereau D, Kelly SE, Eagles C, Charach A, et al. Pharmacologic treatment of attention deficit hyperactivity disorder in adults: A systematic review and network meta-analysis. PLoS one. 2020;15(10):e0240584.

Faraone SV, Po MD, Komolova M, Cortese S. Sleep-Associated Adverse Events During Methylphenidate Treatment of Attention-Deficit/Hyperactivity Disorder: A Meta-Analysis. J Clin Psychiatry. 2019;80(3):18r12210

Ghuman JK, Arnold LE, Anthony BJ. Psychopharmacological and other treatments in preschool children with attention-deficit/hyperactivity disorder: current evidence and practice. J Child Adolesc Psychopharmacol. 2008;18(5):413-47.

Godfrey J. Safety of therapeutic methylphenidate in adults: a systematic review of the evidence. J Psychopharmacol. 2009;23(2):194-205.

Hanwella R, Senanayake M, de Silva V. Comparative efficacy and acceptability of methylphenidate and atomoxetine in treatment of attention deficit hyperactivity disorder in children and adolescents: a meta-analysis. BMC Psychiatry. 2011;11:176.

Hennissen L, Bakker MJ, Banaschewski T, Carucci S, Coghill D, Danckaerts M, et al. Cardiovascular Effects of Stimulant and Non-Stimulant Medication for Children and Adolescents with ADHD: A Systematic Review and Meta-Analysis of Trials of Methylphenidate, Amphetamines and Atomoxetine. CNS Drugs. 2017;31(3):199-215.

Holmskov M, Storebo OJ, Moreira-Maia CR, Ramstad E, Magnusson FL, Krogh HB, et al. Gastrointestinal adverse events during methylphenidate treatment of children and adolescents with attention deficit hyperactivity disorder: A systematic review with meta-analysis and Trial Sequential Analysis of randomised clinical trials. PLoS one. 2017;12(6):e0178187.

Joseph A, Ayyagari R, Xie M, Cai S, Xie J, Huss M, et al. Comparative efficacy and safety of attention-deficit/hyperactivity disorder pharmacotherapies, including guanfacine extended release: a mixed treatment comparison. Eur Child Adolesc Psychiatry. 2017;26(8):875-97.

Koren G, Barer Y, Ornoy A. Fetal safety of methylphenidate-A scoping review and meta analysis. Reprod Toxicol. 2020;93:230-4.

Kortekaas-Rijlaarsdam AF, Luman M, Sonuga-Barke E, Oosterlaan J. Does methylphenidate improve academic performance? A systematic review and meta-analysis. Eur Child Adolesc Psychiatry. 2019;28(2):155-64.

Lan Y, Zhang LL, Luo R. Attention deficit hyperactivity disorder in children: comparative efficacy of traditional Chinese medicine and methylphenidate. J Int Med Res. 2009;37(3):939-48.

Lenzi F, Cortese S, Harris J, Masi G. Pharmacotherapy of emotional dysregulation in adults with ADHD: A systematic review and meta-analysis. Neurosci Biobehav Rev. 2018;84:359-67.

Liang EF, Lim SZ, Tam WW, Ho CS, Zhang MW, McIntyre RS, et al. The Effect of Methylphenidate and Atomoxetine on Heart Rate and Systolic Blood Pressure in Young People and Adults with Attention-Deficit Hyperactivity Disorder (ADHD): Systematic Review, Meta-Analysis, and Meta-Regression. Int J of Environ Res Public health. 2018;15(8):1789

Liu H, Feng W, Zhang D. Association of ADHD medications with the risk of cardiovascular diseases: a meta-analysis. Eur Child Adolesc Psychiatry. 2019;28(10):1283-93.

2Liu Q, Zhang H, Fang Q, Qin L. Comparative efficacy and safety of methylphenidate and atomoxetine for attention-deficit hyperactivity disorder in children and adolescents: Meta-analysis based on head-to-head trials. J Clin Exp Neuropsychol. 2017;39(9):854-65.

Lv X-Z, Shu Z, Zhang Y-W, Wu S-S, Zhan S-Y. Effectiveness and safety of methylphenidate and atomoxetine for attention deficit hyperactivity disorder: a systematic review. Transl Pediatr. 2012;1(1):47-53.

Maneeton N, Maneeton B, Intaprasert S, Woottiluk P. A systematic review of randomized controlled trials of bupropion versus methylphenidate in the treatment of attention-deficit/hyperactivity disorder. Neuropsychiatr Dis Treat. 2014;10:1439-49.

Padilha SCOS, Virtuoso S, Tonin F, S. a, Borba HHL, Pontarolo R. Efficacy and safety of drugs for attention deficit hyperactivity disorder in children and adolescents: a network meta-analysis. Eur Child Adolesc Psychiatry. 2018;27(10):1335-45.

Pievsky MA, McGrath RE. Neurocognitive effects of methylphenidate in adults with attention-deficit/hyperactivity disorder: A meta-analysis. Neurosci Biobehav Rev. 2018;90:447-55.

Pozzi M, Carnovale C, Peeters GGAM, Gentili M, Antoniazzi S, Radice S, et al. Adverse drug events related to mood and emotion in paediatric patients treated for ADHD: A meta-analysis. J Affect Disord. 2018;238:161-78.

Prasad V, Brogan E, Mulvaney C, Grainge M, Stanton W, Sayal K. How effective are drug treatments for children with ADHD at improving on-task behaviour and academic achievement in the school classroom? A systematic review and meta-analysis. Eur Child Adolesc Psychiatry. 2013;22(4):203-16.

Rezaei G, am, Hosseini SA, Akbari Sari A, Olyaeemanesh A, Lotfi MH, et al. Comparative efficacy of methylphenidate and atomoxetine in the treatment of attention deficit hyperactivity disorder in children and adolescents: A systematic review and meta-analysis. Med J Islam Repub Iran. 2016;30:325.

Rodrigues R, Lai MC, Beswick A, Gorman DA, Anagnostou E, Szatmari P, et al. Practitioner Review: Pharmacological treatment of attention-deficit/hyperactivity disorder symptoms in children and youth with autism spectrum disorder: a systematic review and meta-analysis. J Child Psychol Psychiatry. 2021;62(6):680-700.

Roskell NS, Setyawan J, Zimovetz EA, Hodgkins P. Systematic evidence synthesis of treatments for ADHD in children and adolescents: indirect treatment comparisons of lisdexamfetamine with methylphenidate and atomoxetine. Curr Med Res Opin. 2014;30(8):1673-85.

Storebo OJ, Pedersen N, Ramstad E, Kielsholm ML, Nielsen SS, Krogh HB, et al. Methylphenidate for attention deficit hyperactivity disorder (ADHD) in children and adolescents - assessment of adverse events in non-randomised studies. Cochrane Database Syst Rev. 2018;5:CD012069.

Stuhec M, Lukic P, Locatelli I. Efficacy, Acceptability, and Tolerability of Lisdexamfetamine, Mixed Amphetamine Salts, Methylphenidate, and Modafinil in the Treatment of Attention-Deficit Hyperactivity Disorder in Adults: A Systematic Review and Meta-analysis. Ann Pharmacother. 2019;53(2):121-33.

Sun C-K, Tseng P-T, Wu C-K, Li D-J, Chen T-Y, Stubbs B, et al. Therapeutic effects of methylphenidate for attention-deficit/hyperactivity disorder in children with borderline intellectual functioning or intellectual disability: A systematic review and meta-analysis. Sci Rep.2019;9(1):15908.

Tamminga HGH, Reneman L, Huizenga HM, Geurts HM. Effects of methylphenidate on executive functioning in attention-deficit/hyperactivity disorder across the lifespan: a meta-regression analysis. Psychol Med. 2016;46(9):1791-807.

Tarrant N, Roy M, Deb S, Odedra S, Retzer A, Roy A. The effectiveness of methylphenidate in the management of Attention Deficit Hyperactivity Disorder (ADHD) in people with intellectual disabilities: A systematic review. Res Dev Disabil. 2018;83:217-32.

Van der Oord S, Prins PJM, Oosterlaan J, Emmelkamp PMG. Efficacy of methylphenidate, psychosocial treatments and their combination in school-aged children with ADHD: a meta-analysis. Clin Psychol Rev. 2008;28(5):783-800.

Villas-Boas CB, Chierrito D, Fernandez-Llimos F, Tonin FS, Sanches ACC. Pharmacological treatment of attention-deficit hyperactivity disorder comorbid with an anxiety disorder: a systematic review. Int Clin Psychopharmacol. 2019;34(2):57-64.

Yan L, Wang S, Yuan Y, Zhang J. Effects of neurofeedback versus methylphenidate for the treatment of ADHD: systematic review and meta-analysis of head-to-head trials. Evid Based Ment Health. 2019;22(3):111-7.
